# Virus-Induced Aggregates in Infected Cells

**DOI:** 10.3390/v4102218

**Published:** 2012-10-17

**Authors:** Adi Moshe, Rena Gorovits

**Affiliations:** Institute of Plant Sciences and Genetics in Agriculture and the Otto Warburg Minerva Center for Agricultural Biotechnology, The Robert H. Smith Faculty of Agriculture, Food and Environment, The Hebrew University of Jerusalem, Rehovot 76100, Israel

**Keywords:** plant virus, mammalian virus, aggregation

## Abstract

During infection, many viruses induce cellular remodeling, resulting in the formation of insoluble aggregates/inclusions, usually containing viral structural proteins. Identification of aggregates has become a useful diagnostic tool for certain viral infections. There is wide variety of viral aggregates, which differ by their location, size, content and putative function. The role of aggregation in the context of a specific virus is often poorly understood, especially in the case of plant viruses. The aggregates are utilized by viruses to house a large complex of proteins of both viral and host origin to promote virus replication, translation, intra- and intercellular transportation. Aggregated structures may protect viral functional complexes from the cellular degradation machinery. Alternatively, the activation of host defense mechanisms may involve sequestration of virus components in aggregates, followed by their neutralization as toxic for the host cell. The diversity of virus-induced aggregates in mammalian and plant cells is the subject of this review.

## 1. Introduction

Increasing evidence suggests that the assembly of many mammalian viruses occurs at specific intracellular sites, which have been termed “virus factories”. The ultrastructure of the factories has been determined for a number of RNA and large DNA viruses that assemble in the cytoplasm, at the microtubule organizing center (MTOC) [1,2,3]. In the case of DNA viruses that replicate in the nucleus, the identity and structure of virus assembly sites are not clear, likely due to the complexity and the dynamic nature of the nuclear architecture. Virus inclusions in nuclei are often formed in promyelocytic leukemia nuclear bodies and in nuclear aggresomes [4]. In plant cells, both RNA and DNA viruses are associated with large inclusions detected in the cytoplasm and nucleus, however, their role in virus propagation or oppositely in virus restraint is less investigated than in infected mammalian cells.

In general, mammalian and plant viruses make use of aggregates as scaffolds for anchoring the replication complex, increasing the local concentration of viral and host components required for replication and assembly, and shielding the process of replication from host defense. Alternatively, these aggregates may be part of an innate cellular response that recognizes virus components and targets them for storage and degradation. To understand the aggregation processes accompanying virus infection, it is important to discover the origin of the cellular components that gives rise to the virus‑induced inclusions and smaller aggregates, and to identify the molecular motors that are involved in their trafficking from the site of origin to the final destination. Virus aggregates often result in rearrangement of cellular membrane compartments and/or cytoskeleton. The functions of these organelles are carefully regulated in cells. Changes in cellular architecture may constitute responses to the stress associated with virus infection. Throughout this review we suggest that the line that separates viral aggregates as storage of dead-end material from a functional viral factory is rather artificial. Viruses may target key stages in the regulatory pathways that control organelle structure and function to generate sites that are essential for replication and assembly. The same structures can be associated with cellular defenses against infection and cell stress. Given the co-evolution of viruses with their host cells, changes in cell structure induced during infection are likely to involve a combination of the two strategies.

## 2. Virus Factories Are the Sites of Accumulation and Assembly of DNA and RNA Viruses in Mammalian Cells

Numerous viruses assemble and replicate in large insoluble inclusion bodies. In the case of mammalian viruses, these inclusions, called “virus factories” or “viroplasm”, are generally localized near the MTOC and maintained by dynein microtubule motor proteins (reviewed in [5]). Virus factories concentrate viral components needed for the genome replication and morphogenesis of new virus particles. The same structures also contain cellular chaperones/heat shock proteins (HSPs), proteases, and the elements of 26S proteasome degradation machinery, which might enable cellular protective mechanisms to target viral compounds for degradation. In this regard, virus factories are functionally comparable with the cellular aggresomes, where aggregated toxic proteins are immobilized for subsequent proteasomal or autophagic degradation [6]. Hence, for many viruses, transport to the MTOC for storage and eventual degradation is a means to protect cells from infection. Lately, two newly discovered cellular compartments named the “ER-associated compartment” (ERAC) and the “juxtanuclear quality control compartment” (JUNQ) have been shown to have common features with aggresomes. In addition, distinct cytoplasmic inclusions, coined ‘insoluble protein deposit’ (IPODs), result from the accumulation of aggregation-prone mostly non-ubiquitinated substrates that are sequestered to protect the cell from the consequences of their potential toxicity [7]. Given that aggresomes, JUNQ, IPODs can be found in yeast and mammalian cells, it is reasonable to hypothesize that the mechanisms promoting cellular aggregation and inclusion formation are conserved across kingdoms. The same statement could be applied for virus-induced aggregation even though analogy between virus aggregates, JUNQ and/or IPOD has not been demonstrated.

**Figure 1:**
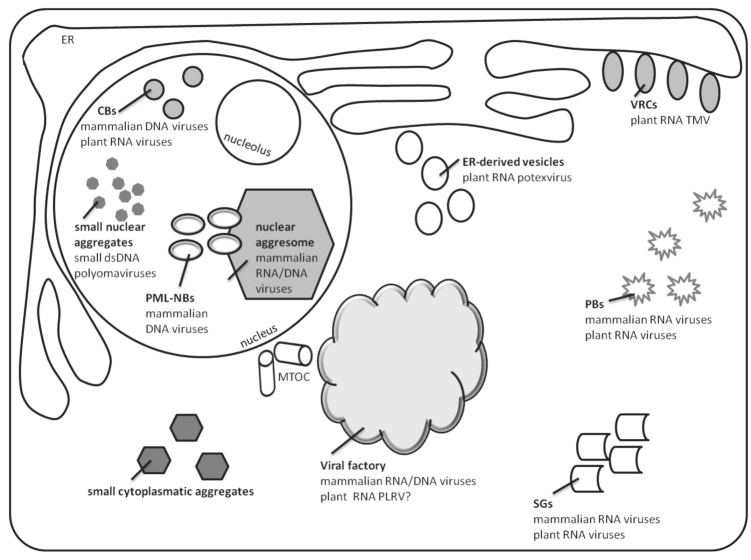
Cellular targets of viral infection induced aggregation. The scheme represents the different types of virus-induced aggregates discussed in the current review, as well as their cellular localization. Typical examples for each type are presented.

Once in the cell, viruses may appear to the host as foreign or misfolded proteins, stimulating an aggresome response. Many viral core particles have a size (60 to 100 nm) similar to the aggregates that are transported to aggresomes by dynein motors [8]. In the case of large viruses such as Poxviruses and African swine fever virus (collectively named nucleocytoplasmic large DNA viruses, or NCLDV), replication and assembly take place in viral factories that contain viral DNA and structural proteins and resemble pericentriolar aggresomes. This raises the possibility that the aggresome pathway is used by these viruses to generate sites for replication and assembly [2,4]. MTOC-dependent development of virus factories was found also for mammalian RNA viruses of types I/togaviruses around lysosomes, type II/flaviviruses, RNA viruses III/bunyaviruses, coronaviruses, and arteriviruses (reviewed in [1]). In general, cytoplasmic virus factories are considered as gathering points for coordinated genome replication and capsid protein assembly into virions. At the same time, these subcellular domains could protect host cells from toxic viral proteins degraded by 26S proteosome or via autophagy pathways [4] (see scheme in Figure 1). 

## 3. Sites of Accumulation and Assembly of RNA Viruses in Plant Cells

MTOCs are not found in plant cells. Therefore, viral proteins are not gathered according to the aggresome-like pathway for microtubule dependent aggregation of toxic or misfolded proteins. Nonetheless, several studies demonstrated the possible involvement of an aggresome-like pathway in movement protein (MP) accumulation during RNA virus infections. Plasmid encoded protein MP17 of *Potato leaf roll virus* (PLRV) developed large aggregates in cells treated with the 26S proteosome inhibitor clasto-lactacystin B-lactone [9]. *Tobacco mosaic virus* (TMV) 30-kDa MP was shown to become polyubiquitinated during virus infection and, subsequently, to enter the 26S proteasome degradation pathway [10]. TMV infection mobilized perinuclear and cytoplasmic endoplasmic reticulum (ER) membranes to form virus replication complexes (VRCs) where the TMV MP was localized (Figure 1). VRCs moved to adjacent cells through plasmodesmata as large bodies; VRCs contained the components necessary to initiate a rapid spread of infection. VRC cell-to-cell spread was blocked by inhibitors of actin and myosin. The proposed model implied that TMV cell-to-cell spread was achieved by VRCs dispersed throughout the cortical region of the cell [11].

The association of positive-stranded RNA viruses’ replicating machineries with intracellular membranes is a feature common in animals, plants and insects (reviewed in [3]). Intracellular membranes are used by different viruses to anchor their replication complexes. For example, ER appears to be re-configured in such a way that the surface of the membrane is used as a scaffold, where viral replication complexes are juxtaposed with capsid proteins specifically delivered to these locations [12]. Complex interactions between viral and host proteins in these structures, different from mammalian virus factories, served for the regulation of *Potato virus X* (PVX) multiplication. PVX replicase was detected in large membrane bound containers that developed from the ER (approximately 375 nm in diameter) together with the virus MP TGBp3 [13]. TGBp3 caused the increased expression of the transcription factor bZIP60, which functions as an ER resident sensor of stress [14]. Consequently, the expression of ER resident chaperones, such as BiP, PDI, and SKP1, which is a component of SCF ubiquitin ligase complexes, are affected by PVX TGBp3, while silencing bZIP60 expression in protoplasts greatly inhibits PVX replication [15]. In general, plant positive strand RNA viruses are associated with membranes originating from the ER and from organelles. These membranous complexes protect the virus replication and translation machineries from degradation by proteases and nucleases, and also protect the viral RNA from silencing (reviewed in [16]).

## 4. Viral Components Localized in Nuclear Aggregates

Herpes simplex virus type-1, Adenovirus type 5 (Ad5), and SV40 accumulate at nuclear sites named promyelocytic leukemia nuclear bodies (PML, previously termed ND10) [17,18,19,20]. In uninfected cells, the nucleus contains five to thirty PML/ND10 bodies, which may serve as scaffolds for mobilizing a variety of proteins involved in transcriptional regulation, chromatin organization, and DNA repair. Many DNA viruses appropriate the cellular DNA repair proteins for their replication [21] and use PML/ND10s for capsid assembly. In the infected cells, virus inclusions also appear as subnuclear structures called nuclear aggresomes [22]. They contain proteasome subunits, ubiquitin, and molecular chaperones; they specialize in the containment and/or removal of protein aggregates. Nuclear aggresomes lie adjacent to PML/ND10s and to sites of virus replication (Figure 1). In nuclear aggresomes, misfolded proteins are not discarded by autophagy, which makes them favorite sites for virus replication in the nucleus [23]. Recent studies revealed that nuclear aggresomes could also mobilize cellular proteins that inhibited virus replication [24].

Evidence has accumulated showing that not only DNA viruses, but also RNA viruses belonging to several different families relate to PML/ND10s. Human foamy virus, Human T-cell leukemia virus type 1, Human immunodeficiency virus type 1 and many other mammalian RNA viruses were found to interact with the subnuclear structure PML/ND10s [25]. It is important to note that the major ND10 components constitute host factors with antiviral activities. Almost all herpesviruses, for which replication has been linked to ND10, have evolved regulatory proteins that are capable of inactivating ND10 components or disturbing the integrity of the whole subnuclear structure [25]. Moreover, an enhanced infectivity of human cytomegalovirus was observed in the absence of either one of the basic ND10 components [26]. Herpesviruses are able to induce additional types of nuclear inclusion bodies. The tegument proteins VP22 and VP13/14 are localized in aggregates that align closely but do not overlap with PML/ND10s [27]. It is not known whether these different structures relate to each other, whether they are homogenous accumulations of the individual herpesvirus protein(s), or whether they are simply dead-end aggregates of viral proteins. Different aggregates are induced by small dsDNA polyomaviruses (infectious pathogens of mammals and birds) [28]. The polyomavirus-specific structures/virus factories are not dependent on the presence of the PML protein, and virus replication is not affected in knockout mice [29]. The role of PML/ND10s in polyomavirus replication is complex and PML/ND10associated proteins may provide the necessary architectural foundation for the structures that appear to be polyomavirus factories.

Cajal bodies (CBs) could be considered as a part of the nuclear inclusion family (Figure 1). CBs are structures present in the nucleus of plant and animal cells. They contain small nuclear/nucleolar ribonucleoprotein particles (snRNP, snoRNP) and other proteins such as collin and fibrillarin. They are involved in maturation of spliceosomal snRNPs and snoRNPs [30]. Infection of HeLa cells by Ad5, a double-stranded DNA virus, leads to CB fragmentation into smaller foci, organized in ring structures termed rosettes. CB rosettes localize to the periphery of viral E2A-72K-containing replication centers and disappear at later stages of infection, suggesting a role in adenovirus late gene expression [31]. In plants, the ssRNA Groundnut rosette virus (GRV) protein ORF3 is responsible for long distance movement via the phloem. GRV-ORF 3 is produced in the cytoplasm, moves into the nucleus where it recognizes CBs, and, consequently, forms multiple CB-like structures (CBLs) and promotes their fusion with the nucleolus. It interacts with host proteins, one of which is fibrillarin, normally found in CB and nucleolus. These complexes migrate to the cytoplasm, where fibrillarin, ORF3 and viral RNA accumulate to form viral filamentous ribonucleoprotein (RNPs) cytoplasmatic inclusions that protect viral RNA from degradation. These RNPs cytoplasmatic aggregates are able to move through the phloem [32].

## 5. Presence of Viral Components in Cytoplasmic Small and Intermediate Aggregates

Viruses often disrupt host cell gene expression. On the other hand, cellular anti-viral response may involve inhibition of viral gene expression via RNA silencing or translational arrest. Degradation, inhibition and storage of mRNA and mRNA-protein complexes (mRNP) are important for cellular homeostasis. While translation of mRNPs takes place in polyribosomes, degradation and storage often occurs in cytoplasmic aggregates [33]. Hence it is expected that viruses interact with stress-related cytoplasmic RNA aggregates, which are important elements of anti-viral defense and at the same time are attractive targets for viral countermeasures [34].

### 5.1. Processing Bodies

Processing bodies (PBs) are cytoplasmic foci in eukaryotic cells that are involved in mRNA decapping, degradation and storage. They contain RNPs, decapping proteins and proteins involved in RNA-induced silencing such as Argonaute (AGO) [35]. Plant PBs have many proteins also found in yeast and mammalian PBs. In Arabidopsis, PBs contain the decapping proteins AtDCP1 and AtDCP2 [36], and the cytoplasmic component of AGO [37]. Mammalian PBs were shown to contain a cellular protein named Moloney leukemia virus 10 (MOV10) [38]. The overexpression of MOV10 reduced HIV infectivity by interrupting early stages of post-entry replication, while MOV10 silencing decreased HIV infectivity. Therefore, it has been suggested that a basic level of the PB machinery is needed for HIV-1 RNA processing and assembly; however, conversely, an increased expression of the PB component MOV10 restricts viral replication [38]. For some plant viruses, accumulation of viral components in PBs may serve as viral replication sites. For example, *Brome mosaic virus* RNAs accumulate in PBs, confirming the importance of PBs in the formation of replication complexes [39].

### 5.2. Cytoplasmic Stress Granules

In mammals, upon a large diversity of stress including viral infection, cells inhibit translation by converting active translation initiation complexes into inactive mRNPs. These complexes are shuttled to cytoplasmic stress granules (SG) for storage (Figure 1). Indeed, active viral infection is not commonly seen if SGs are present, suggesting a role for SGs in inhibition of viral infection. It appears that there are different mechanisms for SG formation, but commonly, SG development is triggered by problems in the initiation of protein translation. PBs may promote SG assembly, and mRNP and other proteins may shuttle between the two structures [34,40]. Many viruses are known for their ability to block SG formation or, alternatively, induce SG disassembly, thereby allowing rapid translation of viral mRNAs. For example, Junin virus blocks SG formation by interrupting the phosphorylation of eIF2 [41]. Some viruses appear to mediate SG response to create virus replication/translation sites. Upon infection of Vaccinia virus, cytoplasmic aggregates emerge and share some properties of SGs, such as the SG marker eIF4E. They do not contain stalled mRNPs but contain viral mRNA. These aggregates are in close proximity to viral replication factories and may form a site for translation separated from replication [42].

In plants, SGs were first observed in the cytoplasm of tomato cells upon heat stress and were termed heat shock granules (HSGs) [43]. In addition to mRNP complexes, HSGs contain heat stress‑induced proteins belonging to the HSP20 family [44]. HSG-like structures may play a role in the RNA-dependent RNA polymerase 6 (RDR6)-mediated siRNA pathway. Accordingly, RDR6‑mediated dsRNA is produced in cytoplasmic suppressor of gene silencing 3 (SGS3)/RDR6 bodies, separated from PBs [45]. Under stress conditions that trigger SGs formation, SGS3/RDR6 bodies co-localize with SGs markers such as eIF4E, suggesting that these structures function as SGs [46]. p2 protein of Rice stripe virus (RSV) was found to be localized in SGS3/RDR6-bodies [47]. SGS3/RDR6-bodies play a role in the multiplication of the plant DNA geminivirus Tomato yellow leaf curl virus (TYLCV). TYLCV V2 protein was shown to be a suppressor of plant RNA silencing [48,49]. V2 interacts with SGS3 in SGS3/RDR6 bodies, impairing SGS3 function in the RNA silencing pathway, resulting in suppression of RNA silencing and enabling viral infection [49].

## 6. Aggregates Induced by Plant DNA Viruses

**Figure 2:**
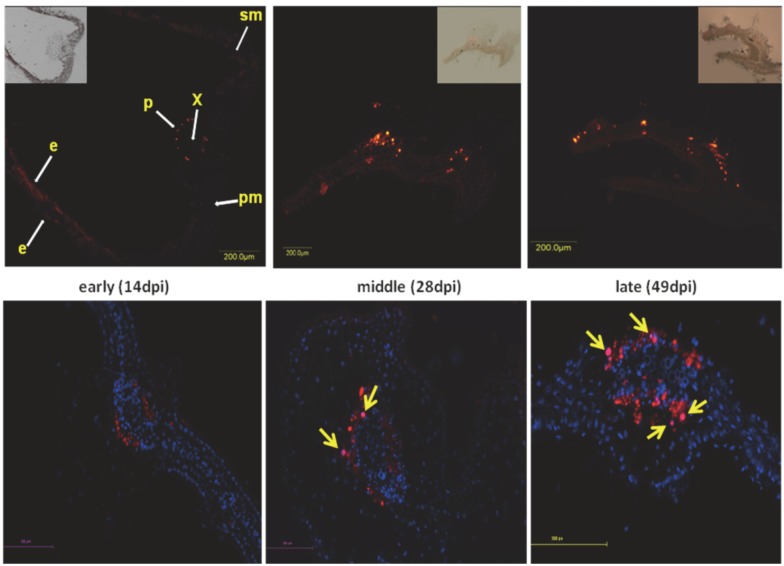
Localization of coat protein (CP) in cross-sections of midribs of infected leaves from susceptible tomatoes. A primary anti-CP antibody and Cy3-labeled secondary antibody (appears as red) were used for fluorescence microscopy. Nuclei are DAPI stained and appear as blue. CP localized in nuclei appears as pink (arrows). Inserts: composite image of fluorescent and transmission microscope settings, e: epidermis, p: phloem, pm: palisade mesophyll, sm: spongy mesophyll, x: xylem. CP associated fluorescence 14 (early), 28 (middle), and 49 (late) days after inoculation (dpi). Bars: 100 µm (upper panel) and 100, 200 µm (lower panel).

The appearance of large inclusion bodies containing virions in geminivirus-infected plants is a characteristic known for many years [50]. The kinetics of formation of DNA virus-related aggregates/inclusion bodies of different sizes and its relation to pathogenesis is the subject of recent investigation. The geminivirus *Indian cassava mosaic virus* AV2 protein implicated in viral movement was shown to form cytoplasmic and nuclear inclusion bodies [51]. Similar inclusions were generated by *Abutilon mosaic virus* [52]. Other plant DNA viruses also form aggregates of different sizes. The caulimovirus *Cauliflower mosaic virus* (CaMV) firstly produced small electron-dense inclusion bodies (EDIBs), which were later exported to a single massive ELIB in each infected cell [53,54,55]. CaMV ELIB’s formation was microtubule-dependent, even though disruption of microtubules by oryzalin did not totally abolish ELIB development [55]. Despite the obvious resemblance of ELIBs formation to that of aggresomes, e.g., microtubule-dependent formation and the “one-per-cell” distribution, ELIBs differ from virus factories because MTOC is absent in plant cells. EDIBs, but not ELIBs, contained CaMV multifunctional protein p6, virus particles and the virion-binding protein pIII. The occurrence of ELIBs was shown to be essential for successful transmission of CaMV by their aphid vector, but was not required for the other viral functions. CaMV mutants that did not form ELIBs were fully infectious [56]. It must be emphasized that the functions of viral inclusions other then virus factories is poorly understood, especially in plants. TYLCV induce aggregates of various size in phloem-associated cells of infected tomato leaves. At early stages of infection, immunodetection of TYLCV coat protein (CP) under the fluorescent microscope showed discrete punctate spots in the cytoplasm. At the later stages, signals of increasing size localized first in cytoplasm then in the nucleus (Figure 2). TYLCV genomic dsDNA replicative form together with CP-DNA complexes were found exclusively in the nuclear aggregates [57]. Moreover, the nuclear inclusion contained infectious particles that could be transmitted by the insect whitefly vector, causing the TYLCV disease in new tomato plants. Several cellular proteins, such as HSP70/HSC70, HSP100, and ubiquitin were detected in the large cytoplasmic and nuclear CP-inclusions [58]. In tomato plants resistant to the virus, the formation of large CP aggregates is delayed and most aggregates are of small size [57]. The molecular basis of natural TYLCV resistance is unknown (reviewed in [59]), however the responses to virus infection of resistant and suscaptible tomato plants at the level of metabolites and proteins patterns are significantly different [60]. In the field, the timing of infection of susceptible seedlings is critical: Deleterious effects of TYLCV (symptoms and arrest of growth) are preeminent when infection occurs during the first three weeks after planting; if infection is delayed, the plants will develop almost normally and will yield [61]. Resistant tomatoes may initiate a protective mechanism which leads to the accumulation of sequestering units in which the virus CP is captured in small/midsized aggregates by host compounds, disabling its capacity to participate in the formation of large inclusions in cytoplasm and consequently in nuclei to develop new virions. Such delay of viral spread allows the plant to grow enough to sustain pathogenesis.

The similarity between TYLCV cytoplasmic large inclusions with perinuclear virus factories or similarity of virion-containing nuclear inclusions with nuclear aggresomes or PML/ND10s is debatable. The absence of MTOC in plants, a required virus factory’s component, has been discussed already; PML/ND10s have not been detected so far in plant cells. At present, we are unable to name the TYLCV-induced inclusions, but their abundance (especially of nuclear aggregates) correlates with efficient virus multiplication. Alternatively, the maintenance of small aggregates containing TYLCV CP is a characteristic of TYLCV-resistant tomatoes [57].

## 7. Discussion

For a long time, electron microscopy studies revealed virus particles arranged in aggregates of different sizes in infected cells. In the current review, we have summarized the multiplicity of virus‑induced aggregates, their function, localization, size and content.

Virus-induced aggregation in plants is much less known than aggregation of mammalian viruses, even though cylindrical inclusions induced by the potyvirus Tobacco etch virus [62], and crystalline arrays caused by TMV [63], were described decades ago. Moreover, the appearance of viral aggregates has been widely used to diagnose viral diseases (see “Extension plant disease clinic”, University of Florida [64]). Another typical characteristic of plant virus invasion known for a long time is the alteration of the morphology of host organelles and membranes, for example by Cowpea chlorotic mottle virus [65] and Cymbidium ringspot virus [66]. In some instances, tubules containing virus-like particles were identified in or near the cell walls of infected cells (by Cowpea mosaic virus, for example [67]). Virus-induced membrane structures, mostly shown for plant RNA viruses, house the RNA replication complex and may be compared with virus factories in infected mammalian cell [3,9], even though plant cells lack MTOC, the most characteristic component of virus factories. Interestingly, these structures were identified as a degrading center for a plant virus movement protein, and thus may be involved in the viral cycle. Recent studies in plant virology emphasize the absolute requirement for the formation of virus inclusions or virus factories for successful virus multiplication (reviewed in [68]). The exact name of certain structures does not seem to be as important as the definition of their role in the virus cycle. For example, in the case of the plant DNA virus CaMV, small multiple aggregates (EDIBs) showed characteristics of virus factories, while single large cytoplasmic aggregates (ELIBs) resembled aggresomal structures and were shown to be important for aphid transmission, but not for CaMV infection in the plant cell [55]. Furthermore, CaMV replication and accumulation in EDIBs were not dependent on microtubule cytoskeleton functioning in contrast to known mammalian virus factories [4].

Virus-induced aggregates play a dual role in virus propagation in the infected cells. The recruitment of host cellular proteins into cytoplasmic and nuclear inclusions to facilitate virus replication has been described for many viruses (see above); on the other hand, the same viruses could be captured in cellular protective aggregative structures. For example, replication sites of DNA Vaccinia virus and ASFV can be targeted by SG components and Mx proteins, respectively [42,69]. Mx proteins are interferon (IFN)-induced members of the dynamin superfamily of large GTPases. In general, Mx GTPases appear to detect viral infection by sensing nucleocapsid-like structures. As a consequence, these viral components are trapped and sorted to locations where they become unavailable for the generation of new virus particles. Mouse Mx1 and human MxA proteins aggregate into punctate granula in the nucleus or cytoplasm, respectively, of IFN-treated cells. The aggregation of Mx proteins prevents their rapid degradation and provides a storage structure from which active molecules can be recruited for prolonged periods of time [70]. In these cases, virus induced aggregations play a protection role. The other well-defined example is the antiviral defense mechanism of PML/ND10 bodies [25]. PML/ND10 components are constitutively expressed, allowing immediate antiviral activity of these molecules unlike interferon-induced antiviral properties of Mx proteins.

In the case of TYLCV, two different types of virus-induced aggregates have been described [57]. Large nuclear inclusions contained DNA-CP complexes and infectious particles were transmitted to test plants by insect vectors, in contrast to cytoplasmic small/midsized aggregates. In cytoplasmic aggregates, TYLCV CP could be trapped and sorted to locations where the main viral protein became unavailable for the generation of new virus particles. Differential states of aggregation could be a part of the plant immune response, reflecting different inclusion types, similar to nuclear aggresome and cytoplasmic IPODs. The plant host quality control machinery recognizes TYLCV compounds as foreign structures and directs them to huge insoluble nuclear inclusions, which the virus uses in its own favor: To house a large complex of proteins of both viral and host origin to promote virus replication and assembly. Alternatively, virus protein(s) is (are) captured and redirected by the plant protective system to SG-like structures or IPOD-like compartments, where they are sequestered and neutralized as protein aggregates. In future research, identification of putative cellular factors within small or large TYLCV aggregates or involved in their arrangement will help to better understand their role in plant protection, and possibly elucidate novel plant cell mechanisms, whether related to IPOD and aggresome assembly. Understanding the phenomenon of virus aggregation and of the response of cells under attack, whether facilitating or inhibiting virus replication, may help to develop novel therapeutic approaches against virus infections in animal and plant cells. Controlling viral diseases of agriculture crops is a major challenge to achieve food security in developed as well as in developing countries. 
